# Common and differential variables of anxiety and depression in adolescence: a nation-wide smartphone-based survey

**DOI:** 10.1186/s13034-024-00793-1

**Published:** 2024-08-17

**Authors:** Martin Weiß, Julian Gutzeit, Rüdiger Pryss, Marcel Romanos, Lorenz Deserno, Grit Hein

**Affiliations:** 1 Department of Psychiatry, Psychosomatic and Psychotherapy, Center of Mental Health, University Hospital Würzburg, Margarete-Höppel-Platz 1, 97080 Würzburg, Germany; 2 Department of Psychology I, University of Würzburg, Würzburg, Germany; 3 Department of Psychology III, University of Würzburg, Würzburg, Germany; 4 Institute of Clinical Epidemiology and Biometry, University of Würzburg, Würzburg, Germany; 5 Institute of Medical Data Science, University Hospital Würzburg, Würzburg, Germany; 6 Department of Child and Adolescent Psychiatry, Psychosomatics and Psychotherapy, Center of Mental Health, University Hospital Würzburg, Würzburg, Germany; 7 Department of Psychiatry and Psychotherapy, Technische Universität Dresden, Dresden, Germany

**Keywords:** Adolescence, Anxiety, Depression, Machine learning, e-Health

## Abstract

**Background:**

Mental health in adolescence is critical in its own right and a predictor of later symptoms of anxiety and depression. To address these mental health challenges, it is crucial to understand the variables linked to anxiety and depression in adolescence.

**Methods:**

Here, we analyzed data of 278 adolescents that were collected in a nation-wide survey provided via a smartphone-based application during the COVID-19 pandemic. We used an elastic net regression machine-learning approach to classify individuals with clinically relevant self-reported symptoms of depression or anxiety. We then identified the most important variables with a combination of permutation feature importance calculation and sequential logistic regressions.

**Results:**

40.30% of participants reported clinically relevant anxiety symptoms, and 37.69% reported depressive symptoms. Both machine-learning models performed well in classifying participants with depressive (AUROC = 0.77) or anxiety (AUROC = 0.83) symptoms and were significantly better than the no-information rate. Feature importance analyses revealed that anxiety and depression in adolescence are commonly related to sleep disturbances (anxiety OR = 2.12, depression OR = 1.80). Differentiating between symptoms, self-reported depression increased with decreasing life satisfaction (OR = 0.43), whereas self-reported anxiety was related to worries about the health of family and friends (OR = 1.98) as well as impulsivity (OR = 2.01).

**Conclusion:**

Our results show that app-based self-reports provide information that can classify symptoms of anxiety and depression in adolescence and thus offer new insights into symptom patterns related to adolescent mental health issues. These findings underscore the potentials of health apps in reaching large cohorts of adolescence and optimize diagnostic and treatment.

**Supplementary Information:**

The online version contains supplementary material available at 10.1186/s13034-024-00793-1.

## Introduction

Anxiety disorders and depressive disorders are among the most prevalent mental health disorders in adolescents worldwide. Pre-pandemic estimates for anxiety and depression indicate that about 11.6% [57] and 12.9% [35] respectively, of adolescents were affected. According to a recent meta-analysis including data from 329,159 children and adolescents ≤ 18 years between January 2020 and December 2023, however, 26% reported symptoms of anxiety and 23% reported symptoms of depression [2]. For distinct countries, the numbers were even higher, as the prevalence during the COVID-19 outbreak for anxiety and depression in Chinese adolescents were 37.4% and 43.7% respectively [61].

In line with these findings, longitudinal studies including data from pre- and during the COVID-19 pandemic report increases in symptoms of depression and anxiety in 13–16 year-old adolescents (e.g., Magson et al. [36, 48]). However, a meta-analysis of 12 longitudinal studies suggest that only depressive symptoms increased significantly, while anxiety symptoms remained stable [7].

Different research reports heterogeneous patterns of predictors that contribute to clinical levels of anxiety and depression in adolescents. For instance, Stewart and colleagues [54] found that in a Scottish sample, self-reported symptoms of anxiety and depression were more likely among older individuals, females, those with current or past mental health support, those needing extra school support, and those experiencing poorer home relationships since the COVID-19 pandemic. In a Jordanian sample, AlAzzam et al. [1] showed that the educational level of both parents, perceived difficulties in online education, sex, and age were related to depression, while only the father’s level of education, difficulties in online education, sex, and age were related to anxiety disorders.

As both disorders can lead to substantial impairments in daily functioning [19], earlier identification and intervention are critical to prevent common chronic and comorbid disease patterns. Further, child and adolescent mental health is one critical predictor of adult mental health (e.g., Otto et al. [41] for review, see Johnson et al. [27]). Therefore, the aim of this study is to identify the predictors of anxiety and depression in a sample of adolescents from a nation-wide survey provided via a smartphone-based application (app) in Germany.

Adolescents participated in the current study without targeted recruitment, as the app was publicly available in the app stores. This approach overcomes several limitations typically associated with (adolescent) mental health research and services (e.g., Bantjes [6]). These limitations include scheduling issues (i.e., adolescents rely on their parents’ scheduling and mobility to participate in research), adolescents’ unwillingness to share personal information with professionals, and their need for autonomy and independence [43]. Serious apps (should) guarantee anonymity, thus helping to overcome stigmatization, and allow users to decide for themselves when and to what extent they provide data about their mental well-being [24]. The use of mental health apps in research thus helps to increase the heterogeneity of samples (e.g., in resource-limited settings, Lehtimaki et al. [32] and by removing geographic barriers, Bührmann et al. [12] and ideally to reach more of those who would need help on the basis of self-diagnosis.

Leveraging these benefits of data retrieved via mental health apps, we used elastic net regressions to classify individuals with levels above the cut-off for anxiety and depression. By variable selection and regularization, this machine-learning method identifies the most important variables associated with anxiety and depression, respectively, and provides robust classifications.

## Methods

### Participants

We included 278 adolescents that provided data via the Corona Health App (129 female, mean age = 15.24, SD = 1.57, range = 12–17). The Corona Health App was initiated by the Mental Health Research Unit of the Robert Koch Institute (RKI), i.e., the German federal agency for public health responsible for disease control and prevention, and the Universities of Würzburg, Ulm and Regensburg (for details, see Beierle et al. [9]). The goal of Corona Health was to monitor mental and physical well-being during the COVID-19 pandemic. Users could download the Corona Health App for free from the Apple Store and from the Google Play store. Thus, there were no specific criteria for recruitment. All participants gave their consent within the app, and without consent the app was closed. After giving consent, participants could select the study they wanted to participate in (in this case, the “Mental Health for Adolescents” module) and were forwarded to a baseline questionnaire. Participants were then able to allow mobile sensing features and schedule follow-up surveys (which is not part of the present analysis). The median duration to fill out the baseline survey was 8:52 min. The present analyses were based on the cross-sectional data collected between July 2020 and September 2022.

### Measurements

The two questionnaires used to assess the dependent variables were the Patient Health Questionnaire-2 for symptoms of depression (PHQ-2, two items, cutoff ≥ 3; Löwe et al. [34] and the Brief Spence Children’s Anxiety Scale for symptoms of anxiety (SCAS-C-8, eight items based on the DSM-5, Cutoff > 6.5; Reardon et al. [46]. The PHQ-2 with a cutoff of ≥ 3 has been shown to be a valid screening of clinically relevant depressive symptoms for 13–17 year olds [47]. The SCAS-C-8 was mainly developed for younger samples, but has also been used for adolescents up to the age of 18 (e.g., Orgilés et al. [40]). The reliabilities of both questionnaires in our sample were Cronbach’s α = 0.79 and α = 0.84, for depression and anxiety, respectively. Apart from quality of life, all other items uniquely assessed an aspect in the dataset (e.g., worries about COVID-19, media usage, going outside, impulsivity, arguments at home/school, prior anxiety and depression disorder, etc.). All items were selected or generated by a multi-professional team of different domain experts (app developers, medical professionals, psychologists, etc.) in a multi-stage process of discussion and several feedback loops [9].

### Analysis

We used elastic net logistic regression, a regularized regression method, to classify adolescents’ above vs. below the cutoffs for clinically relevant symptoms of depression and anxiety. With the elastic net, the number of selected features can be even larger than the sample size, achieving a sparse model [13, 63]. We chose to classify cases and controls (i.e., individuals above and below the cutoff of clinically relevant levels of anxiety and depression) as this might be more helpful for practitioners in clinical practice. For the remaining 60 items, we applied an iterative imputation method based on a random forest using the “missForest” package in *R* (R Core Team 2021) to obtain a data set without missing values [53]. In this way, 25 missing values were imputed in the training data set and 9 missing values in the test data set. These items were then included in our analyses (z-standardized metric or dummy-coded in case of factors). This means that in the depression model, the 60 items plus the anxiety cut-off item were used and in the anxiety model, the 60 items plus the depression cut-off item were used. A list of all included items can be found in the Supplementary Materials. We randomly selected 70% of the data as the training dataset and applied 5-fold repeated cross-validation to train and tune our model over a grid of α and λ hyperparameters, using the *R* package “caret” [29]. First, we refitted the model on the training dataset with the best performing hyperparameters to calculate the final penalized β coefficients. Second, we applied the model to the remaining 30% of the sample, i.e., the test dataset, to estimate model performance (accuracy, area under the receiver operating characteristic curve [AUROC], sensitivity, specificity, positive and negative prediction value, balanced accuracy, and Cohen’s kappa). With this split we aimed for a large enough train data set for adequately tuning hyperparameters while still holding out a large enough test set to achieve a robust model performance evaluation accounting for the imbalanced dataset (see Table 1). We conducted separate variable importance analyses for depression and anxiety models using permutation importance on the test data set. This technique involves repeatedly permuting (100 permutations in our case) features and evaluating model performance with each variable in a non-informative context. The importance of each feature is quantified by calculating the decrease in model performance (AUROC) resulting from the lack of information provided by the permuted feature [3]. To identify the most important variables we computed multiple logistic regressions sequentially including all predictors in order of the calculated importance determined by the elastic net regression starting with only one (the most important) predictor and ending with all predictors (including the least important predictor last). We then selected the model with the lowest Bayesian information criterion (BIC), a measure indicating goodness of fit while simultaneously penalizing complexity of the model and thus addressing overfitting. We then tested the best performing models with the reduced feature space on the test data set to investigate the generalizability and robustness of our findings.

## Results

### Sample

Classification of participants with clinically relevant depressive or anxiety symptoms by gender are described in Table 1. The comorbidity of depressive and anxiety symptoms in our study was 22.8%. The reliabilities (Cronbach’s α) for PHQ2 and SCAS-8 are 0.79 and 0.83, respectively. The training and test data set did not differ significantly regarding demographic characteristics (see Supplementary material, Table S2).

**Table 1:** Prevalence of clinically significant symptoms of depression (PHQ2 ≥ 3) and anxiety (SCAS-8 > 6.5) and gender

	Male	Female	Total
No symptoms of depression	95 (68.35%)	72 (55.81%)	167 (62.31%)
Symptoms of depression	44 (31.65%)	57 (44.19%)	101 (37.69%)
No symptoms of anxiety	96 (69.04%)	64 (49.61%)	160 (59.70%)
Symptoms of Anxiety	43 (30.94%)	65 (50.39%)	108 (40.30%)

Absolute numbers and relative numbers for each gender group in parentheses

### Depression

In summary, the classification model for symptoms of depression had an overall accuracy of 71.60% with a 95% confidence interval of [60.50%, 81.07%] and an AUROC of 0.77. The accuracy was significantly better than the no-information rate (NIR = 61.73%, *p* = 0.041). It performed well in classifying healthy participants (specificity = 90.00%) but showed a low sensitivity of 41.94%. Despite that, it was able to classify individuals with clinically relevant symptoms of depression (positive prediction value = 72.22%) and healthy participants (negative prediction value = 71.43%). Overall, the model had a kappa value of 0.347 (considered “fair” according to Landis and Koch [31] and a balanced accuracy of 65.97%.

**Fig. 1:**
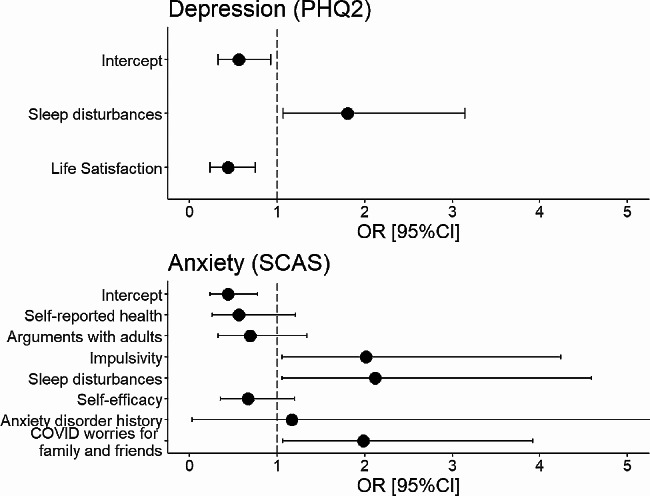
Odds ratios and 95% CIs of logistic regression models with the most important predictors determined by BIC

The top two predictors identified by the BIC for the classification of depressive symptoms were *sleep disturbances* and *life satisfaction* (Fig. 1, upper panel).

The logistic regression with these two predictors as independent variables and depression scores as dependent variable on the test data set was significant. (χ² = 19.68, *p* < 0.001, Nagelkerke R² = 0.293) and both predictors had a significant effect on the odds of being depressed (see Table 2).

**Table 2:** Coefficients and odds-ratios of the logistic regression on depression with top two predictors of the elastic net regression

**Predictor**	**Estimate**	**SE**	**z**	*p*	OR [95%CI]
(**Intercept**)	**-0.58**	**0.26**	**-2.21**	**0.027**	**0.55 [0.33; 0.93]**
**Sleep disturbances**	**0.59**	**0.27**	**2.16**	**0.031**	**1.80 [1.07; 3.14]**
**Life satisfaction**	**-0.83**	**0.29**	**-2.83**	**0.005**	**0.43 [0.24; 0.75]**

Significant predictors are shown in bold

### Anxiety

The classification model for symptoms of anxiety showed an overall accuracy of 76.54% with a 95% confidence interval of [65.82%, 82.52%] and an AUROC of 0.83. The difference in information rate compared to the no-information rate was significant (NIR: 64.20%, *p =* 0.012). The model had a sensitivity of 65.52% and a specificity of 82.69%. It successfully classified between healthy individuals and individuals with anxiety (negative prediction value = 81.13%; positive prediction value = 67.86% indicating a higher false positive rate than false negative rate). Overall, the model had a moderate performance, as indicated by its kappa value of 0.486 and its balanced accuracy of 74.10%.

The top seven predictors selected with BIC for the classification of anxiety were *self-reported health*,* arguments with adults*,* impulsivity*, *sleep disturbances*, *self-efficacy*, *anxiety disorder history* and *COVID worries for family and friends* (Fig. 1, lower panel).

The logistic regression with these seven predictors as independent variables and anxiety as dependent variable on the test data set was significant (χ² = 30.42, *p* < 0.001, Nagelkerke R² = 0.430). However, only predictors *impulsivity*,* sleep disturbances*, and *COVID worries for family and friends* had a significant effect on the odds of suffering from anxiety. All coefficients can be seen in Table 3.

## Discussion

In this study, we analyzed self-reported data of adolescents collected in their everyday lives via the RKI Corona Health App between July 2020 and September 2022 to find associations between relevant variables and symptoms of depression and anxiety. We computed two elastic net regression models to classify healthy individuals and individuals with symptoms of depression or symptoms of anxiety, respectively. Both models achieved significantly higher accuracies in classifying cases then the non-information rates and classified most cases correctly. We conducted a variable importance analysis, identifying significant predictors for depression and anxiety symptoms with common and distinct profiles.

**Table 3:** Coefficients and odds-ratios of the logistic regression on anxiety with top seven predictors of the elastic net regression

Predictor	Estimate	SE	z	*p*	OR [95%CI]
**(Intercept)**	**− 0.83**	**0.30**	**− 2.71**	**0.007**	**0.44 [0.23; 0.78]**
Self-reported health	− 0.57	0.39	− 1.47	0.143	0.56 [0.26; 1.21]
Arguments with adults	− 0.37	0.36	− 1.03	0.303	0.69 [0.33; 1.34]
**Impulsivity**	**0.70**	**0.35**	**2.01**	**0.045**	**2.01 [1.06; 4.24]**
**Sleep disturbances**	**0.75**	**0.37**	**2.03**	**0.042**	**2.12 [1.06; 4.59]**
Self-efficacy	− 0.41	0.31	− 1.33	0.184	0.67 [0.35; 1.20]
Anxiety disorder history	0.15	1.67	0.09	0.927	1.16 [0.03, 42.25]
**COVID worries for family and friends**	**0.68**	**0.33**	**2.08**	**0.037**	**1.98 [1.06; 3.91]**

Significant predictors are shown in bold

A clear advantage of this analysis is the assessment of mental health via smartphone app. this gives many people the opportunity to take part in such a survey who might otherwise have experienced certain geographical, scheduling or motivational barriers to participating in a conventional survey. As a matter of fact, we found comparably high prevalence of symptoms of anxiety and depression in our sample. 37.69% of all participants were classified with clinically significant symptoms of depression and 40.30% with clinically significant symptoms of anxiety. These prevalences are noticeably higher than reported in recent literature (e.g., 23.3% for anxiety disorders in German adolescents, Niermann et al. [39] 11.5% for depression in German adolescents Scheiner et al. [50]. This could imply that more affected people were reached by the survey via the app. However, direct comparisons between our prevalence rates and these reported rates must be interpreted cautiously. In our study, anxiety and depressive symptoms were assessed using self-report screenings in a self-selected sample. In both reported studies, the samples were larger and randomly drawn from the population. Mental disorders were assessed either with multiple, longer scales than ours [50] or with a standardized interview [39]. Thus, due to the psychometric properties of our measurements, we might have over-detected clinically relevant symptoms in our sample compared to those in these studies (see Limitations).

In both symptom constellations sleep disturbances had the largest odds ratio, i.e., the largest effect size, in the reduced models. This could indicate various things. First, there could be a non-causal relation between sleep-disturbances, symptoms of anxiety, and symptoms of depression in our sample explained by a fourth variable. For instance, family disorganization (i.e., lack of structure and routine within the home) has been associated with sleep disturbance, anxiety disorders [58], and an increased risk for depression [22]. During the pandemic, families were confronted with increased strain and commotion due to several COVID-19 related stressors, such as physical and mental health concerns, economic stress, intensified relationships, and conflicts [59]. However, symptoms of anxiety, symptoms of depression, and sleep disturbances could also be more directly related to each other. The finding that sleep disturbances in adolescents are associated with both symptoms of depression and anxiety is well documented [21]. Sleep disturbances are a common symptom of both depression and generalized anxiety disorder according to the *Diagnostic and Statistical Manual of Mental Disorders* (5th ed.; DSM–5; APA, [4]. There is a high comorbidity of depression and anxiety disorders, ranging up to 50% in community samples [19] and 22.8% symptom comorbidity in our study. Since the present study only examines cross-sectional data, it is not possible to say clearly whether sleep disorders are the cause or consequence of symptoms of anxiety or depression, or whether they arise primarily in the comorbidity of these symptom clusters. However, a large body of research has shown that preceding sleep disturbances are an important risk factor for both disorders (e.g., [10, 14, 17, 21]). Potential mechanisms could be a decrease of anterior white matter tracts and connectivity in the fronto-limbic network caused by sleep disturbance [26]. These regions are associated with the control and regulation of negative emotions. Impaired processing in these regions might lead to psychological processes associated both with anxiety and affective disorders [10]. In conclusion, while it is difficult to safely determine the direction of the triadic association link between sleep disturbance, anxiety symptoms, and depressive symptoms from this cross-sectional data, we conclude that sleep disturbances have a high importance for detecting or diagnosing symptoms of anxiety and depression.

Apart from the predictor overlap of sleep disturbances, the models differ noticeably regarding their most important variables. In the reduced depression model, only life satisfaction was a significant predictor besides sleep disturbances, showing an inverse relation with depressive symptoms. There is evidence that higher life satisfaction is an indicator of an adaptive and positive psychosocial functioning which is a protective factor against emotional deficits, such as symptoms of depression [20, 44]. Although we do not want to draw any causal conclusions on the relationship between life satisfaction and depression, the directionality (i.e., life satisfaction as regressor for depression as criterion) and the inverse relationship is in line with previous research (e.g., Tang et al. [55]). Except for sleep disturbances and (reduced) life satisfaction, there were no more significant predictors for depressive symptoms in the reduced model.

Interestingly, impulsivity was the predictor with the second largest effect for self-reported anxiety symptoms. Past research has reported an inverse relation between anxiety disorders and impulsivity [5, 8]. However, more recent research has also shown positive associations between impulsivity and anxiety disorders among adolescents (e.g., Moustafa et al., [37]). This relationship could arise indirectly through the presence of another mental disorder. For instance, almost every second child diagnosed with ADHD—a mental disorder that is associated with increased impulsivity—suffers from a comorbid mood disorder [62]. This assumption is supported by evidence using cross-sectional and longitudinal psychometric networks showing that ADHD is reciprocally associated with internalizing symptoms via potential bridging symptoms primarily related to anxiety symptoms [52]. There is also evidence that Cyclothymia might also be a common factor of increased impulsivity and anxiety [42]. Last, while anxiety in mood disorders might increase impulsive behaviors related to suicide [16, 51], we doubt that this is the primary driver of the observed relationship in our dataset. However, it is conceivable that heightened anxiety may specifically increase impulsivity in areas of daily life associated with anxiety.

The third significant aspect of symptoms of anxiety is COVID worries for family and friends. In the different phases of the pandemic, adolescents experienced health-related worry regarding their own health as well as the health of their families and peers—but also worries related to their social lives (for review, see Guessoum et al. [23]. It has been shown that such worries had a direct effect on general anxiety [38]. Independent of the pandemic context, worry was found to be a stable construct that has been linked to anxiety disorders throughout adolescence [45]. Worrying is also bidirectionally related to sleep disturbances [56], another important predictor for anxiety in our model (see above).

More than one in five participants showed comorbid depressive and anxiety symptoms. We did not train a third model to identify this subgroup. However, we would expect sleep disturbances to be one of the most important predictors, as it had high predictive value in both separate models. We would also expect life satisfaction to be strongly decreased for these patients, as it was strongly associated with depressive symptoms and has been shown to be the case for patients suffering from comorbid anxiety and depression [15].

In conclusion, the models classifying depressive and anxiety symptoms show a clear overlap regarding sleep disturbances but differ noticeably in other predictors, namely life satisfaction, impulsivity, and COVID-worries. These findings make an important contribution to the differential diagnosis of both disorders. Our analyses cannot clearly determine whether the significant predictors are risk factors, symptoms, or other relevant variables of the respective syndromes. However, due to the predictor overlap of sleep disorders of both symptom clusters, one could conclude that sleep quality is of great importance for the general prevention of mental illness in adolescents [60].

### Limitations

This study focused solely on the baseline measurement of our app-based survey, analyzing cross-sectional data. While longitudinal data from follow-up surveys were collected, we excluded these from the current analysis. Our decision to concentrate on cross-sectional data was driven by the need for a sufficient sample size to conduct robust machine learning analyses. The lower follow-up rate of participants made the longitudinal data less suitable for this purpose. However, future research could explore the temporal development of depressive and anxiety symptoms using the follow-up data, potentially identifying preceding risk and resilience factors.

Even though the classification model for depressive symptoms performed significantly better than the no information rate, it had a rather low sensitivity. This indicates difficulties in reliably detecting participants with clinically relevant depressive symptoms above the cut-off. One potential reason for this might be the psychometric characteristics of the used outcome. There is, for instance, evidence that the PHQ-2 tends to have low specificity in detecting clinically relevant depressive symptoms [33]. This might have led to an over-detection of individuals with “truly” clinically relevant depressive symptoms, as is also evident in our rather high prevalence rate. Thus, the model might have had difficulties in identifying robust associations between several relevant variables, symptom constellations, and depressive symptoms, as the prevalence of the latter might have been overestimated (though this could also be attributed to self-selection bias, discussed below). The rationale for choosing this short screening tool was to find a balance between survey brevity and accuracy. Shorter surveys tend to have better response rates [49], and the aim of the original study was to assess a wide range of different constructs [9]. For future research, however, a combination of the PHQ-2 with the PHQ-9 might yield better accuracy in identifying the criterion of depressive symptoms [33]. Another alternative would be to address the imbalance between depressive symptoms (two items) and symptoms of anxiety (eight items) by assessing general distress using the Kessler Psychological Distress Scale (K10; Kessler et al. [28]). The K10 includes 10 questions about emotional states, serves as a brief screening tool to identify levels of distress and has been used effectively in adolescents (e.g., Boyes et al. [11]). Generally, many of the variables were assessed by only one item. While this again had clear benefits regarding survey length, it limits generalizability and the ability to capture nuances in the constructs. Although the outcome measure for the anxiety model had also only moderate accuracy [46], the performance seemed to be less affected by this than the depression model. This might indicate that the training fit was better due to slightly higher prevalence of cases with relevant anxiety symptoms then depression symptoms. Another explanation could be that the relations between some potential risk-factors and symptom patterns of depression were more complex and harder to detect for the elastic net regressions, especially compared to the anxiety model.

In conclusion, both models showed significant improvements in classifying outcomes over respective no-information rates. The performance of both models, which is satisfactory in some areas only, must be considered in the context of the partially imprecise outcome measures and can still be regarded as accurate in general.

Generally, we found a comparably high prevalence rate of mental health problems in our sample. This might have been due to a self-selection bias in our sample: As the Corona Health App was advertised in the German media and participation in the survey was voluntary, one could argue that individuals who were already feeling unwell or were heavily worried by the pandemic were more motivated to participate in the study. Moreover, one might wonder who downloaded the app completely voluntarily, and who was advised or encouraged by their parents. 14.71% of participants reported being in psychotherapy at the time of the survey, a notably higher rate than the German national average of adolescents (1.4% of all German children/adolescents, highest rate of 2.5% among 15- to 19-year-olds; Jaite et al. [25]. Thus, worried parents might have encouraged their children to seek help or partake in scientific studies regarding mental health, leading to a potentially un-representative sample. This potential bias, however, may have actually enhanced our analyses and models. By providing a more balanced sample than the general population, which typically includes far more healthy individuals than those with anxiety or depressive symptoms, we potentially improved accuracy. Highly imbalanced data can skew model classification, as the majority class tends to be overrepresented and over-classified [30]. Some researchers deliberately overrepresent the minority class—in our case, participants with clinically relevant symptoms—by under-sampling the majority or over-sampling the minority [18]. Consequently, we believe the classification analyses yielded robust results, despite the possibility of self-selection bias.

## Conclusion

In conclusion, our data gathered from individuals across Germany based on a self-downloaded App give novel insights into symptom constellations associated with mental health problems in adolescents. The overlapping and distinct symptoms of anxiety and depression can help informing prevention programs aiming at early detection of mental health issues. Employing advanced data-driven methodologies on extensive datasets not only advances scientific knowledge but also paves the way for more effective and personalized strategies to foster resilient mental health among today’s youth.

## Electronic supplementary material

Supplementary Material 1.

## Data Availability

The data and scripts that support the findings of this study are openly available at https://osf.io/fxnuv/.
