# Correction to: Common and differential variables of anxiety and depression in adolescence: a nation-wide smartphone-based survey

**DOI:** 10.1186/s13034-025-00877-6

**Published:** 2025-03-20

**Authors:** Martin Weiß, Julian Gutzeit, Rüdiger Pryss, Marcel Romanos, Lorenz Deserno, Grit Hein

**Affiliations:** 1 Department of Psychiatry, Psychosomatic and Psychotherapy, Center of Mental Health, University Hospital Würzburg, Margarete-Höppel-Platz 1, 97080 Würzburg, Germany; 2 Department of Psychology I, University of Würzburg, Würzburg, Germany; 3 Department of Psychology III, University of Würzburg, Würzburg, Germany; 4 Institute of Clinical Epidemiology and Biometry, University of Würzburg, Würzburg, Germany; 5 Institute of Medical Data Science, University Hospital Würzburg, Würzburg, Germany; 6 Department of Child and Adolescent Psychiatry, Psychosomatics and Psychotherapy, Center of Mental Health, University Hospital Würzburg, Würzburg, Germany; 7 Department of Psychiatry and Psychotherapy, Technische Universität Dresden, Dresden, Germany

**Correction to: Child and Adolescent Psychiatry and Mental Health (2024) 18:103** 10.1186/s13034-024-00793-1

In the original publication of this article [1], the affiliation details for the authors were incorrectly linked in the author group. The incorrect and correct affiliations are listed in this correction article. The original article has been updated.

**Incorrect**:

Martin Weiß^1,7*†^, Julian Gutzeit^1,2†^, Rüdiger Pryss^3,4^, Marcel Romanos^5^, Lorenz Deserno^5,6^ and Grit Hein^1^.

^1^Department of Psychiatry, Psychosomatic and Psychotherapy, Center of Mental Health, University Hospital Würzburg, Margarete-Höppel-Platz 1, 97,080 Würzburg, Germany.

^2^Department of Psychology III, University of Würzburg, Würzburg, Germany.

^5^Department of Child and Adolescent Psychiatry, Psychosomatics and Psychotherapy, Center of Mental Health, University Hospital Würzburg, Würzburg, Germany.

^3^Institute of Clinical Epidemiology and Biometry, University of Würzburg, Würzburg, Germany.

^4^Institute of Medical Data Science, University Hospital Würzburg, Würzburg, Germany.

^6^Department of Psychiatry and Psychotherapy, Technische Universität Dresden, Dresden, Germany.

^7^Department of Psychology I, University of Würzburg, Würzburg, Germany.

**Correct:**

Martin Weiß^1,2*†^, Julian Gutzeit^1,3†^, Rüdiger Pryss^4,5^, Marcel Romanos^6^, Lorenz Deserno^6,7^ and Grit Hein^1^.

^1^Department of Psychiatry, Psychosomatic and Psychotherapy, Center of Mental Health, University Hospital Würzburg, Margarete-Höppel-Platz 1, 97,080 Würzburg, Germany.

^2^Department of Psychology I, University of Würzburg, Würzburg, Germany.

^3^Department of Psychology III, University of Würzburg, Würzburg, Germany.

^4^Institute of Clinical Epidemiology and Biometry, University of Würzburg, Würzburg, Germany.

^5^Institute of Medical Data Science, University Hospital Würzburg, Würzburg, Germany.

^6^Department of Child and Adolescent Psychiatry, Psychosomatics and Psychotherapy, Center of Mental Health, University Hospital Würzburg, Würzburg, Germany.

^7^Department of Psychiatry and Psychotherapy, Technische Universität Dresden, Dresden, Germany.
