# Supplementary material for: Common and differential variables of anxiety and depression in adolescence: a nation-wide smartphone-based survey
Source: Child Adolesc Psychiatry Ment Health. 2024 Aug 17;18:103. doi: 10.1186/s13034-024-00793-1 (PMC11330155; doi:10.1186/s13034-024-00793-1)
Supplement: Supplementary file 1 — Supplementary Material 1. [file 13034_2024_793_MOESM1_ESM.pdf]

Supplementary Material

Common and differential variables of anxiety and depression in adolescence – A nation-wide smartphone-based survey

Table S1. All variables included in both analyses. Depicted are item name in data set, a short description of the item and in questions from the original questionnaire.

item_name	item	question
**depression**	depression (outcome variable)	
**anxiety**	anxiety (outcome variable)	
**kj_age**	Age	How old are you (in years)?
**kj_sex**	Gender	What is your gender?
**kj_house1**	Persons living in household	How many persons currently live in your household?
**kj_house2**	Adolescents living in household	How many of these are children or adolescents (i.e. under the age of 18 years)?
**kj_school3**	Opinion regarding homeschooling	What is your opinion/feeling about being schooled at home / about working remotely from home compared to regular school/work.
**kj_ses**	Socioeconomic status	Answer this question by marking a number from 1 to 10.<br>At "10" are those families with the most money, with the best education and the best jobs.<br>At "1" are those families who are poorest, have poor education and the least jobs or no jobs. Now think of your family. At which number would your family be?
**kj_life**	Life satisfaction	Answer this question by marking a number from (0) to (10). (10) signifies the best imaginable life. (0) signifies the worst imaginable life. What number would your life currently be?
**kj_health**	Self-reported health	In general, how would you describe your health?
**kj_psyth**	Currently in psychotherapy	Are you in psychotherapy?
**kj_smoke**	Smoker	Do you smoke?
**kj_alc**	Alcohol consumption	Do you drink alcohol?
**kj_adhd1**	Attention difficulties	Do you find it difficult to focus attention on tasks (e.g. homework) or on play activities (e.g. a board game)?
**kj_adhd2**	Easily distracted	Are you easily distracted during tasks that require attention?
**kj_adhd3**	Difficulties staying seated	Is it difficult for you to stay seated when you are expected to?
**kj_adhd4**	Impulsivity	Do you act impulsively without thinking about consequences?
**kj_odd1**	Losing temper	Do you easily get upset and lose your temper?
**kj_odd2**	Arguments with adults	Do you often argue and talk back with your parents or teachers?
**kj_odd3**	Disobedience at home	Do you defy or disobey rules at home, at school or at other places?

**kj_selfeff**	Self-efficacy	Is the following statement true for you? If I am in trouble, I can usually think of a solution.
**kj_contact2**	Change in interactions (COVID)	Since the coronavirus pandemic how have your interactions with others outside your home changed?
**kj_contact3**	Attitude towards tele-contact	Do you think that contact via phone or digital media can replace personal contact?
**kj_media1**	Time on media since COVID	Since the coronavirus pandemic how much time do you spend on media (e.g. tv, video games, web surfing, social media)?
**kj_famclim1**	Family mood since COVID	Since the coronavirus pandemic how has the general mood in your family changed?
**kj_famarg1**	Family quarrels since COVID	Since the coronavirus pandemic how often does your family fight?
**kj_cv_fam**	COVID infections in family	Is somebody in your family infected with the coronavirus?
**kj_cvad**	Deceased family member (COVID)	Has a member of your family or somebody you know died due to an infection with the coronavirus?
**kj_school4**	Visiting School	Which statement is correct in regard to school/training/work? 1 = I currently attend school / go to work, 2 = I am schooled at home / I am working remotely from home,3 = Answers do not apply
**kj_restr_day**	Attitude towards COVID restrictions	How do you feel about the restrictions due to the Corona-pandemic? Is your daily life ... [5 point Likert scale from “Much more stressful“ to “Much more pleasant”]
**kj_restr_out**	Times going out	How often do you currently go out (to school, work, take a walk, shopping etc.)?
**kj_sport**	Times exercising	How many times have you exercised (e.g. running, ball sports, etc.) in the past week?
**kj_olfac**	Smell-loss	Have you experienced a loss of smell or taste?
**kj_famclim2**	Family general mood	What was the general mood in your family?
**kj_famarg2**	General quarrels family	Have there been fights in your family?
**kj_viol**	Physical violence in family	Have you or somebody in your family experienced physical violence?
**kj_anx2**	Worries catching COVID	Have you worried about catching the coronavirus.
**kj_anx3**	Worries infecting someone	Have you worried about infecting someone else with the coronavirus.
**kj_media2**	Daily media consumption	How many hours have you spent on average per day on digital media (e.g. tv, video games, web surfing, social media)?
**kj_phq_sleep**	Sleep disturbances	Trouble falling asleep, staying asleep, or sleeping too much?
**kj_liv_wohnung**	Living in apartment	Which of these describes your home? (Multiple selections possible)
**kj_liv_haus**	Living in house	
**kj_liv_balkon**	Apartment/house has a balcony	

**kj_liv_terasse**	Apartment/house has a terrace	
**kj_liv_garten**	Apartment/house has a garden	
**kj_contact_nocontact**	No contact to friends	Since the coronavirus pandemic how do you stay connected with your friends? You may mark more than one answer.
**kj_contact_tel**	Contact to friends via telephone	
**kj_contact_text**	Contact to friends via texting	
**kj_contact_video**	Contact to friends via video chat	
**kj_contact_pers**	Personal contact to friends	
**kj_school1_schule**	Student	Are you attending school or are you pursuing other activities/occupations?
**kj_school1_ausbildung**	Trainee	
**kj_school2_hauptschulabschluss**	Secondary school certificate 1	Which degree are you planning to complete (or have you completed)?
**kj_school2_realschulabschluss**	Secondary school certificate 2	
**kj_school2_abitur**	A-levels	
**kj_diag_keine**	No prior psychiatric diagnosis	Have you ever been told by a doctor or therapist that you have a mental illness?
**kj_diag_angst**	Prior anxiety disorder	
**kj_diag_depression**	Prior depression	
**kj_diag_autismus**	Prior autism	
**kj_nat_deutsch**	German	What is your nationality?
**kj_cv_inf_no_infection**	No COVID infection	Have you been infected with the coronavirus (proven by a test)?

Table S2. Demographics of training and test data set and statistical comparison.

variable		***test set***	***training set***	***test***
gender	female	39 (30.23%)	90 (69.77%)	p value: 0.998
	male	42 (30.22%)	97 (69.78%)	(Pearson's Chi-squared test)
age	Min / Max	12.0 / 17.0	12.0 / 17.0	p value: 0.779
	Med [IQR]	16.0 [14.0;17.0]	16.0 [14.0;17.0]	(Wilcoxon rank sum test)
	Mean (std)	15.2 (1.6)	15.3 (1.5)	
	N (NA)	81 (0)	187 (0)	
